# Efficacy and Prognostic Factors of Trans-Arterial Chemoembolization Combined With Stereotactic Body Radiation Therapy for BCLC Stage B Hepatocellular Carcinoma

**DOI:** 10.3389/fonc.2021.640461

**Published:** 2021-07-16

**Authors:** Changchen Jiang, Shenghua Jing, Han Zhou, Aomei Li, Xiangnan Qiu, Xixu Zhu, Zetian Shen

**Affiliations:** Department of Radiation Oncology, Jinling Hospital, Medical School of Nanjing University, Nanjing, China

**Keywords:** hepatocellular carcinoma, trans-arterial chemoembolization, CyberKnife, stereotactic body radiation therapy, BCLC B

## Abstract

**Purpose:**

This study aimed to evaluate the efficacy and safety of trans-arterial chemoembolization (TACE) followed by stereotactic body radiation therapy (SBRT) in treating Barcelona Clinic Liver Cancer (BCLC) stage B hepatocellular carcinoma (HCC) not amenable to resection and radiofrequency ablation (RFA).

**Methods:**

From February 2012 to January 2017, a total of 57 BCLC stage B HCC patients who were unsuitable candidates for resection and RFA treated with TACE combined with CyberKnife SBRT were included in this retrospective study. Patients underwent TACE for a median of two times (1–5 times) before SBRT. SBRT prescription doses ranged from 30 Gy to 50 Gy in 3–5 fractions.

**Results:**

The median follow-up time was 42 months. The objective response rate (CR + PR) was 85.9%, and the disease control rate (CR + PR + SD) was 96.5%. The local control (LC) rates were 91.1% and 84.3% at 1 and 2 years, respectively. The 1-, 2-, 3-year overall survival (OS) and the median survival time were 73.2%, 51.4%, 32.4% and 26.6 months, respectively. The 1-, 2-, and 3-year progression-free survival (PFS) were 34.2%, 21.6%, and 9%, respectively, with a median PFS time of 9.7 months. A subgroup analysis was conducted in 32 patients with AFP ≥ 200 ng/ml before TACE. OS was significantly prolonged in those with AFP that decreased by more than 75% than those with AFP that decreased by less than 75% (P = 0.018) after SBRT. The treatment was well tolerated with only one patient (1.8%) developed grade 3 gastrointestinal toxicity, and another patient developed non-classical RILD. In multivariate analysis, tumor length ≥ 10 cm and AFP ≥ 200 ng/ml were independent poor prognostic factors for OS.

**Conclusion:**

The combination of TACE and Cyberknife SBRT showed optimal efficacy with acceptable toxicity for BCLC stage B HCC.

## Introduction

Primary liver cancer is the sixth most commonly diagnosed cancer, and its mortality rate ranks fourth around the world. According to the estimates of GLOBOCAN 2018 statistics produced by the International Agency for Research on Cancer of the World Health Organization, there are about 841,000 new cases and 782,000 deaths due to liver cancer annually. The ratio of death to new cases is as high as 0.9 (1). China is the worst-hit area of primary liver cancer with a 5-year survival rate of around 10% (2). Liver resection and transplantation are the main radical treatments and associated with superior clinical outcome, but liver cancer is difficult to diagnose early and progresses rapidly. Only 15% of patients could receive surgical treatment when diagnosed. For patients with Barcelona Clinic Liver Cancer (BCLC) stage B hepatocellular carcinoma (HCC), trans-arterial chemoembolization (TACE) is the recommended therapy. However, the tumor response rate after TACE and local control (LC) rate for those with tumors larger than 5 cm, multiple intrahepatic lesions, cirrhosis, or portal vein tumor thrombus (PVTT) are still not satisfactory (3, 4). All these data highlight the unmet need of optimizing the loco-regional therapy effect in the management of HCC.

The National Comprehensive Cancer Network (NCCN) guidelines recommended that TACE combined with radiotherapy could improve the LC rate and prolong the survival time of patients with unresectable HCC, which was more effective than TACE and sorafenib (5, 6). However, the role of conventional radiotherapy in HCC has long been overlooked because of the low tolerance of the whole liver to radiation. Delivering high tumoricidal dose without causing radiation-induced liver disease (RILD) and affecting adjacent stomach, duodenum, and other endangered organs is difficult (7). In recent years, with the improvements of radiotherapy technology, SBRT, a highly conformal radiation therapy with high geometric precision and accuracy, can deliver a potent dose to target lesions while reducing the dose to adjacent normal tissues, providing a new therapeutic option for inoperable HCC patients. TACE combined with SBRT might have synergistic effects in the treatment of patients with inoperable HCC (8–11). Theoretically, TACE is well controlled in the tumor center but poorly controlled in the oxygen-rich area around the tumor, whereas SBRT is poorly controlled in the hypoxic area in the large tumor center but has a good curative effect in the oxygen-rich area around the tumor. The combination of the two treatment strategies can compensate for each other’s deficiencies and give full play to their advantages. Thus, in this study, we retrospectively analyzed the clinical outcome of combined CyberKnife SBRT and TACE in the treatment of BCLC stage B HCC. The results are reported as follows.

## Materials and Methods

### Clinical Data

From February 2012 to January 2017, a total of 57 patients with BCLC stage B primary liver cancer who received TACE combined with CyberKnife SBRT treatment in Nanjing Jinling Hospital were included in this retrospective study. Inclusion criteria: 1. Patients were diagnosed as HCC by biopsy, and the imaging manifestations were nodular or lumpy; 2. BCLC stage B, Child–Pugh score A–B7 and ECOG 0–1; 3. Unsuitable for resection, liver transplantation, or local ablation therapies according to the comprehensive assessment of hepatobiliary surgery experts, oncologists, interventional experts, and radiologists; 4. Remaining healthy liver > 700 ml. Exclusion criteria: 1. Portal vein thrombus, lymph node involvement, and extrahepatic metastasis; 2. ECOG ≥ 2; 3. Poor liver function with Child–Pugh score of C; 4. Diffuse liver cancer or nonmeasurable lesion, tumor number ≥ 4; 5. Other life-threatening conditions, such as cardiac ischemia or cerebrovascular accident within the last 6 months.

From February 2012 to January 2017, a total of 57 patients received the combined TACE and CyberKnife SBRT treatment as presented above. In our study, 44 (77.2%) patients were hepatitis B carriers. All patients had BCLC stage B disease. The median tumor size was 8.4 cm (range, 4.5–16.3 cm). None of the patients had previously received any other treatment, and no patient dropped out after TACE. The median number of TACE is 2 (range, 1–5). The CyberKnife SBRT prescription doses ranged from 30 Gy to 50 Gy in 3–5 fractions. The median BED10 was 100 Gy (range, 48–124 Gy). The median interval between TACE and SBRT was 37 days (23–69 days). Baseline patient and tumor characteristics are displayed in **Table 1**.

**Table 1:** Baseline patient and tumor characteristics of the 57 patients.

Item	Cases	Percentage(%) or median
Age		
<65	29	50.9
≥65	28	49.1
Gender		
Male	46	80.7
Female	11	19.3
HBVs Ag		
positive	44	77.2
negative	13	22.8
Pre-TACE AFP (ng/ml)		
<200	25	43.9
≥200	32	56.1
AST(U/L)		
≤40	19	33.3
>40	38	66.7
Total bilirubin (mg/dl)		
≥2	9	84.2
<2	48	15.8
Size of largest lesion		
≥10cm	17	8.4 (4.5-16.3)cm
<10cm	40	
Number of lesions		
1	37	64.9
2-3	20	35.1
Child-Pugh score		
A	47	82.5
B	10	17.5
BED10, Gy		
≥100	30	100 (48-124)
<100	27	
Dose/Fraction		
45-48Gy/3F	14	24.6
40-48Gy/4F	7	12.3
30-50Gy/5F	36	63.2
Number of TACE		
1-2	36	2 (1-5)
>2	21	

### Treatment

TACE: Percutaneous puncture of the femoral artery with Seldinger technique was performed. The catheter was inserted into the hepatic artery or celiac axis under the guidance of DSA. Contrast agent was injected into the catheter to determine the location, size, number, and supply artery of the tumor. After the target lesion is determined, a catheter will be inserted to the feeding artery branch. A mixture of 5–20 ml of lipiodol and chemotherapy agents such as 30–40 mg/m^2^ cisplatinum, 20–40 mg THP, or 500–1500 mg fluorouracil glycosides was slowly injected through the catheter to the tumor site. The amount of the mixture emulsion should depend on the tumor size and arterial blood flow. Thereafter, gelatin sponge particle gelfoam embolization was conducted. Liver enhanced MRI and CT scans were performed 3 to 4 weeks after TACE to evaluate the lesion and short-term efficacy. TACE was repeated 1 to 5 times at intervals of 4 to 6 weeks. The median interval between the last cycle of TACE and CyberKnife SBRT was 37 days (23–69 days).

CyberKnife SBRT: All patients were implanted with 3–6 gold fiducials (size of 6.0 mm × 0.8 mm) within or around the tumor using a CT-guided 19 G needle. A CT plain and enhanced scan was performed about 7 days after the implantation. At this time, edema and local hemorrhage subsided, and the gold fiducials were relatively stable and immobile.

Patients were placed in a supine position and used a vacuum pad to fix the body. CT scanning was conducted, and the slice thickness was 1 mm. Hepatic scans ranged 15 cm above and below the lesions. The gross target volume (GTV) was defined as visible liver tumors at the arterial phase or at the delayed portal phase on the CT or MRI scan. The planning target volume (PTV) was defined as GTV plus a margin of 3 to 5 mm. After expansion, the area of the PTV should be adjusted according to the adjacent critical organs at risk. According to the tumor size, location, and critical organ constrains, patients were treated with prescription dose ranging from 30 Gy to 50 Gy for 3–5 times. Respiratory synchronization and gold standard tracking technology were adopted during the treatment. The prescribed isodose line should encompass >95% of PTV. Dose constraints for critical structures are shown in **Table 2**.

**Table 2:** Dose constraints for critical organs.

Critical organs	Dose constraints (treatment in 3–5 fractions)					
	45-48Gy/3F		40-48Gy/4F		30-50Gy/5F	
	Volume	Dose	Volume	Dose	Volume	Dose
Remaining healthy liver	≥700cc	≤5.7Gy/fx	≥700cc	≤4.8Gy/fx	≥700cc	≤4.2Gy/fx
Stomach	Any point	7.4Gy/fx	Any point	6.8Gy/fx	Any point	6.4Gy/fx
Duodenum	Any point	7.4Gy/fx	Any point	6.8Gy/fx	Any point	6.4Gy/fx
Renal cortex	≥200cc	≤4.8Gy/fx	≥200cc	≤4Gy/fx	≥200cc	≤3.5Gy/fx
Spinal cord	Any point	7.3Gy/fx	Any point	6.5Gy/fx	Any point	6Gy/fx

### Follow-Up and Evaluation

Enhanced upper abdominal CT and MRI were conducted 1 month after the completion of CyberKnife treatment, every 3 months in the first 2 years, and then every 6 months thereafter. According to the Modified Response Evaluation Criteria in Solid Tumors (12), the assessment results were divided into complete response (CR), partial response (PR), progressive disease (PD), and stable disease (SD). LC was defined as no progression within the PTV (patients undergoing liver transplantation or resection after the combined treatment were censored). Progression-free survival (PFS) was defined as the period from the beginning of TACE to the radiological progression of any lesion, appearance of new lesions, or the time at which the patient passed away, whichever occurred first. Overall survival (OS) was defined from the date of starting TACE until death or the final follow-up. Toxicity assessment was based on the National Cancer Institute Common Terminology Criteria for Adverse Events version 4.0. Liver-specific toxicity consists of classic and non-classic RILD. Classic RILD was defined as an increase in alkaline phosphatase exceeding two times the upper limit of normal, and non-classic RILD was defined as an increase in transaminase over five times the upper limit of normal, under the circumstances of lack of disease progression or malignant ascites (13).

### Statistical Analysis

SPSS 22.0 statistical software was used for data analysis. LC, PFS, and OS were calculated using the Kaplan–Meier method. Univariate analyses were used to investigate the relationship between all independent variables and OS. Any factors that were significant in univariate analyses were incorporated into multivariate analyses using the Cox proportional hazards model. P < 0.05 was considered statistically significant.

## Results

### LC

**Figure 1:**
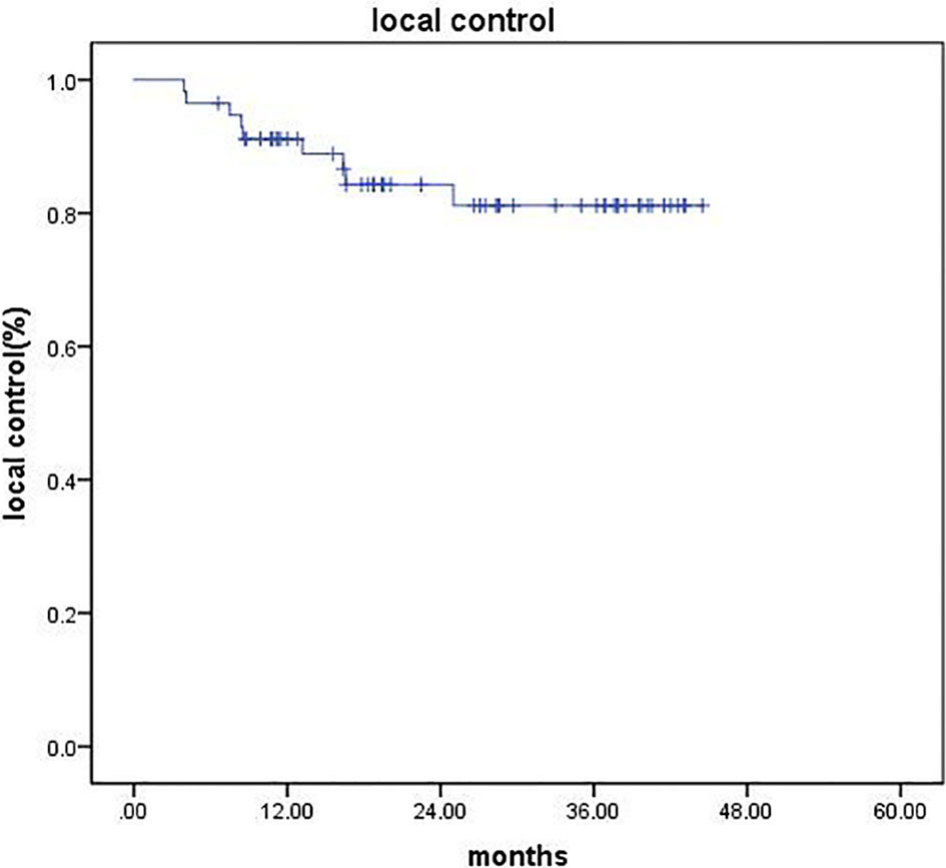
Kaplan–Meier analysis of LC.

After CyberKnife SBRT, liver MRI and/or abdominal CT was evaluated in all patients. CR occurred in 11 patients (19.3%), 38 (66.6%) patients achieved PR, 6 (10.5%) patients showed SD, and 2 (3.5%) cases developed PD. The objective response rate (ORR = CR + PR) was 85.9%. The disease control rate (DCR = CR + PR + SD) was 96.5%. The 1- and 2-year LC rates were 91.1% and 84.3%, respectively (**Figure 1**).

### PFS and OS

**Figure 2:**
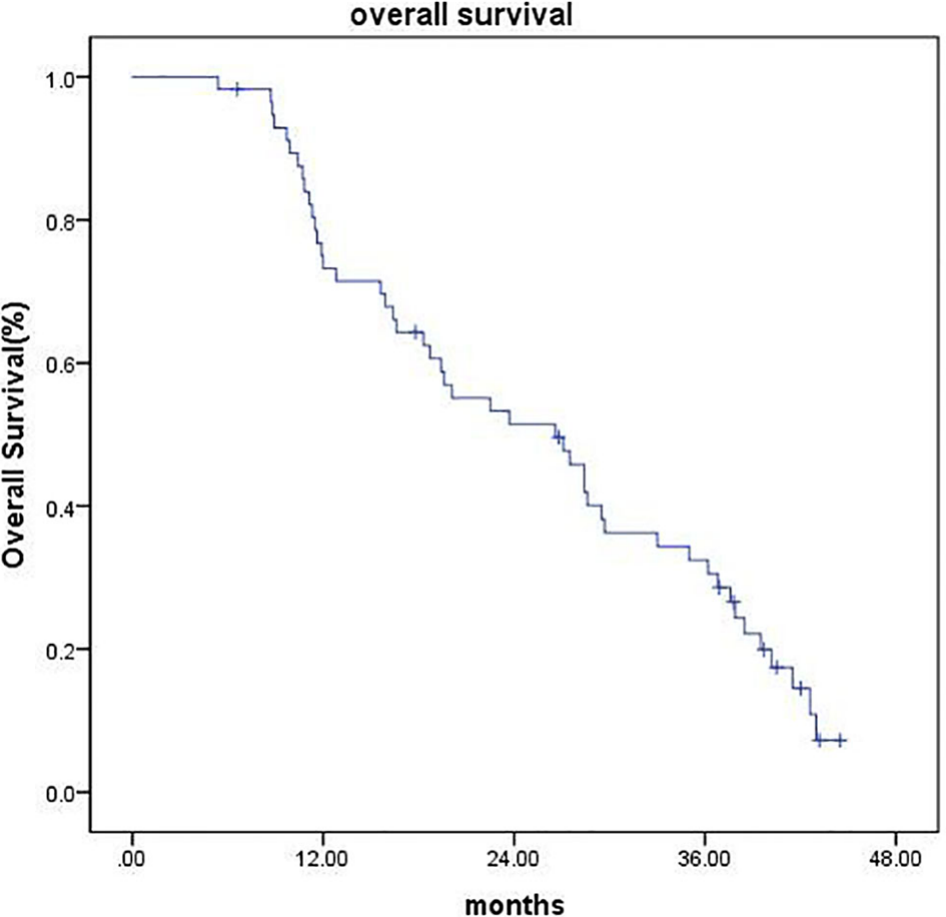
Kaplan–Meier estimates of OS.

**Figure 3:**
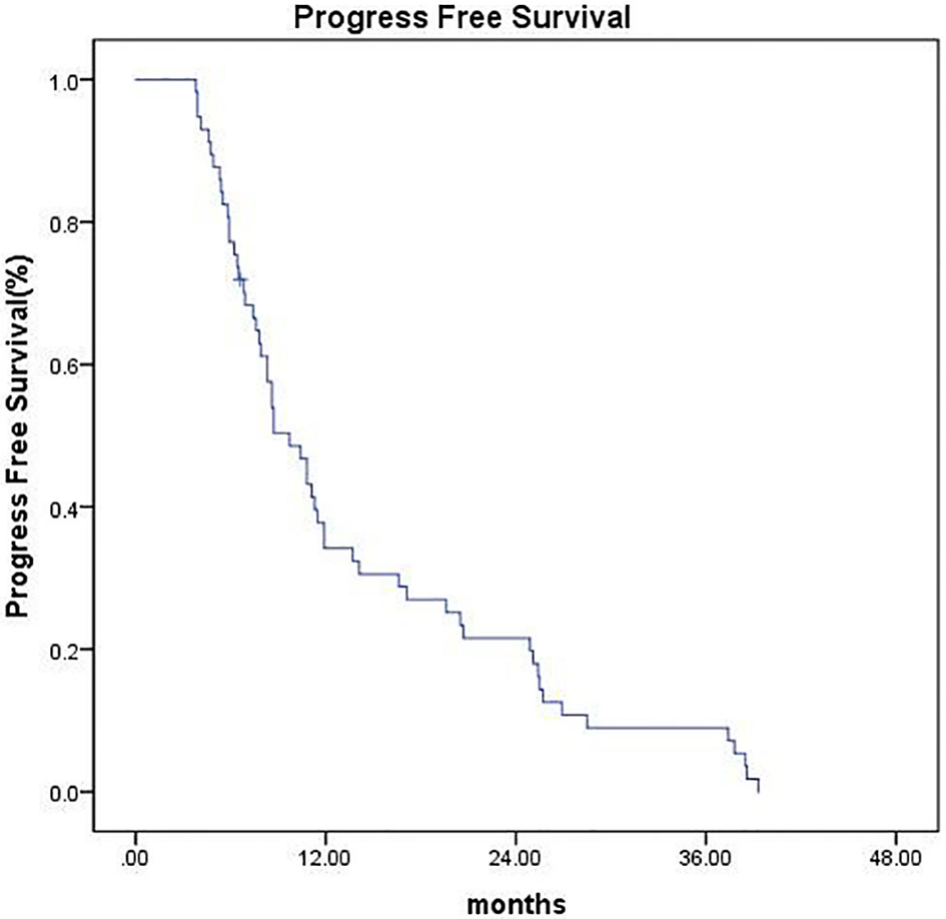
Kaplan–Meier estimates of PFS.

As of the last follow-up date (February 25, 2020), the median follow-up time was 42 months (range, 6.6–44.5 months). Four patients (7.0%) were lost to follow-up at the time of analysis, 1 patient (1.7%) underwent surgical resection, and 1 patient (1.7%) underwent liver transplantation, all of which were recorded as censored. The median OS was 26.6 months (95% CI 18.27–34.92), and the 1-, 2-, and 3-year OS were 73.2%, 51.4%, and 32.4%, respectively (**Figure 2**). The median PFS was 9.7 months (95% CI 7.42–11.97), and the PFS was 34.2%, 21.6%, and 9% at 1, 2, and 3 years, respectively (**Figure 3**).

### Prognostic Factors for OS

The univariate analysis of OS identified five poor prognostic factors, including tumor diameter ≥ 10 cm, multiple nodules, AFP ≥ 200 ng/ml, BED10 < 100 Gy, and TACE times > 2. The significant factors of univariate analysis were enrolled in the multivariate analysis using the Cox proportional hazards regression model. The results showed that tumor diameter ≥ 10 cm and pretreatment AFP ≥ 200 ng/ml were associated with poorer OS (**Table 3**).

**Table 3:** Univariate and multivariate analyses of prognostic factors for OS.

	Univariate HR (95%CI)	P value	Multivariate HR (95%CI)	P value
Gender (male *vs.* female)	0.930 (0.65-1.32)	0.687	—	—
Age (≥65 *vs.* <65)	1.204 (0.67-2.14)	0.529	—	—
HB *vs.* Ag (positive *vs.* negative)	0.883 (0.44-1.74)	0.720	—	—
AFP (≥200 *vs.* <20ng/ml)	0.351 (0.18-0.65)	0.001	0.294 (0.15-0.55)	0.000
AST(>40 *vs.* ≤40U/L)	0.913 (0.66-1.25)	0.573	—	—
Total Bilirubin ( ≥2 *vs.* <2mg/dl)	1.016 (0.67-1.52)	0.938	—	—
Tumor size (≥10 *vs.* <10cm)	0.477 (0.34-0.66)	0.000	0.430 (0.30-0.61)	0.000
Tumor number(single *vs.* Multiple)	0.670 (0.49-0.90)	0.009	—	—
Child-Pugh score (A *vs.* B)	0.736 (0.50-1.06)	0.104	—	—
TACE(1-2 *vs.* >2)	0.697 (0.51-0.93)	0.016	—	—
BED10 (≥100 *vs.* <100Gy)	1.619 (1.19-2.20)	0.002	—	—

### Subgroup Analysis of AFP Determined OS

**Figure 4:**
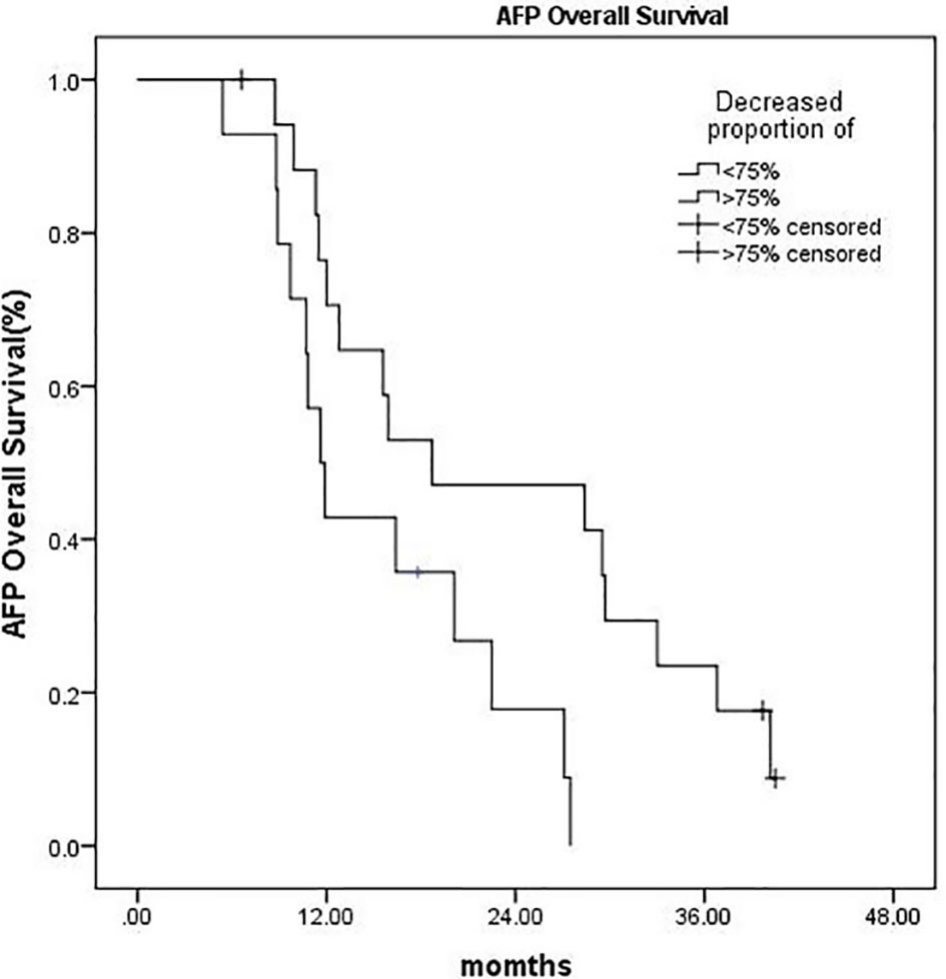
Kaplan–Meier OS curves:AFP decreased more than 75% *vs.* AFP decreased by less than 75% (P = 0.018).

A subgroup analysis was performed on 32 patients with pretreatment AFP ≥ 200 ng/ml. Within 3 months after CyberKnife SBRT, the AFP of 18 patients (56.3%) decreased by more than 75%, and their OS was significantly longer than those whose AFP decreased by less than 75% (P = 0.018) (**Figure 4**).

### Side Effects

Patients mainly experienced grade 1 or 2 fatigue; nausea; vomiting; and hematological toxicity such as anemia, leukopenia, thrombocytopenia, hyperbilirubinemia, and AST elevation. Grade 3 or above adverse events included anemia (3.5%), leukopenia (5.3%), thrombocytopenia (7.1%), AST elevation (5.3%), and hyperbilirubinemia (7%). Three months after the treatment, no patient developed classic RILD, but one patient presented non-classic RILD. One patient developed gastric ulcer 5 months after treatment (**Table 4**). The adverse effects gradually improved after symptomatic treatment. No patients had gastrointestinal perforation, and no treatment-related deaths were found. Before TACE treatment, 82.5% of patients had a Child–Pugh score of A, and 17.5% of patients had a Child–Pugh score of B. However, after 1 month of CyberKnife treatment, 47.4% of patients had a Child–Pugh score of A, and 45.6% of patients had a Child–Pugh score of B.

**Table 4:** Side effects after SBRT.

	Grade1-2, n (%)	Grade 3, n (%)	Grade 4, n (%)	Grade 5, n (%)
Fatigue	19 (33.3)	0 (0)	0 (0)	0 (0)
Nausea	13 (22.8)	0 (0)	0 (0)	0 (0)
Vomiting	8 (14.0)	0 (0)	0 (0)	0 (0)
Gastric ulcer	0 (0)	1 (1.8)	0 (0)	0 (0)
Anemia	10 (17.5)	2 (3.5)	0 (0)	0 (0)
Leukopenia	18 (31.6)	2 (3.5)	1 (1.8)	0 (0)
Thrombocytopenia	26 (45.6)	3 (5.3)	1 (1.8)	0 (0)
Elevation of AST	17 (29.8)	3 (5.3)	0 (0)	0 (0)
Hyperbilirubinemia	11 (19.3)	4 (7)	0 (0)	0 (0)
Non-classic RILD	0 (0)	1 (1.8)	0 (0)	0 (0)

## Discussion

The current guidelines formulated by the NCCN and American Association for the Study of Liver Diseases recommend TACE as the preferred treatment for inoperable HCC. According to the treatment algorithm of the BCLC, TACE is considered as the first-line treatment for intermediate-stage HCC patients who are not suitable for surgical resection or tumor ablation. However, TACE alone demonstrated a dismal CR rate of only 0%–4.8%, and patients cannot achieve satisfactory long-term survival, the 5-year cumulative survival rate of which is only 1%–8% (14). When the tumor size is 5–7 and ≥8 cm, the 2-year OS is only 42% and 0%, respectively (15). Hence, TACE alone might not be sufficient for managing large tumors. Y. Kawamura (16) found that TACE alone can only eradicate 22%–50% of tumor tissues as determined by pathological examination and can minimally eradicate the tumor completely. Thus, TACE was considered as a palliative treatment. To improve the tumor response rate and prolong survival, a large number of studies currently considered the concept of combining TACE with another local therapy, including RFA (17), percutaneous ethanol injection (PEI) (18), and radiotherapy. However, some lesions are unsuitable for RFA and PEI. For example, when the tumor is adjacent to the liver capsule, ablation treatment may lead to rupture of the liver capsule and thus may cause tumor dissemination. Moreover, patients whose tumors are located close to important large blood vessels, bile duct, or gallbladder or deep inside the liver including PVTT are not candidates for percutaneous puncture ablation.

A meta-analysis (5) of 25 studies involving a total of 2,577 patients with unresectable HCC showed the benefits of TACE combined with radiotherapy (mainly three-dimensional conformational radiotherapy) with CR, PR, and 1–5 year OS rates being significantly higher in the combined treatment group than in the chemoembolization alone group (P < 0.001). However, the pooled analysis indicated that the adverse events including gastrointestinal ulcers (OR, 12.80; 95% CI 1.57–104.33), ALT elevation (OR, 2.46; 95% CI 1.30–4.65), and total bilirubin (OR, 2.16; 95% CI 1.05–4.45) were also increased in the combined treatment group. CyberKnife SBRT, which features high-precision radiotherapy, makes up for the deficiency of conventional radiotherapy (19). Several reports indicated that SBRT has a high LC rate and safety in the treatment of liver tumors (8, 19–22). However, the LC rate of SBRT alone in treating increased tumor volume is not satisfactory (10, 23).

In recent years, an increasing number of studies have demonstrated that combination therapy benefits LC compared with monotherapy in the treatment of unresectable HCC (9, 24). In a retrospective study of adjuvant SBRT following TACE in patients who were unsuitable for surgical resection with tumors ≥ 3 cm, Jacob et al. (9) reported that the local recurrence rate was significantly lower in the TACE plus SBRT group compared with the TACE alone group (P = 0.04). Baek Gyu Jun (24) conducted a propensity score matching analysis on HCC patients with tumor size ≤ 5 cm and found that the TACE+SBRT group showed significantly higher 1- and 3-year LC rates (91.1% and 89.9%, respectively) than the TACE alone group (69.9% and 44.8%, respectively; P < 0.001). A phase 2 trial of SBRT as a local salvage treatment after incomplete TACE in patients with HCC < 10 cm was conducted. The results demonstrated that TACE+SBRT treatment achieved promising tumor response rate and LC rate (8). A total of 57 patients with BCLC stage B HCC were enrolled in our study with a median tumor size of 8.4 (range, 4.5–16.3) cm. After the treatment, CR occurred in 11 patients (19.3%). ORR occurred in 49 patients (85.9%), which was superior to 17%–62% in previous studies of TACE alone (25). LC rates at 1 and 2 years were 91.1% and 84.3%, which were comparable to those achieved in previous studies (26, 27).

Clinical data have shown that TACE combined with SBRT in the treatment of unresectable HCC has better long-term survival than TACE or SBRT alone (10, 28). Tiffany CL (28) conducted a propensity score matching analysis including 49 cases of TACE and SBRT combined therapy and 98 cases of TACE alone. The results showed that the median PFS and OS of the combined treatment group were 7.6 and 23.9 months, respectively. The PFS at 1 and 3 years was significantly improved in the combined treatment group (P = 0.012). Meanwhile, the 1- and 3-year OS was also significantly prolonged in the TACE plus SBRT group (P = 0.003). Ting-Shi Su (10) found that in unresectable HCC with tumor size ≥ 5 cm, patients who received TACE followed by SBRT achieved longer OS than those who only received SBRT monotherapy. In our study, the median OS was 26.6 months (95% CI 18.277–34.923). The 1-, 2-, and 3-year OS rates were 73.2%, 51.4%, and 32.4%, respectively. The median PFS was 9.7 months (95% CI 7.427–11.973). The PFS at 1, 2, and 3 years was 34.2%, 21.6%, and 9%, respectively. Previous studies reported that SBRT treatment for unresectable HCC has a 2-year OS of 34%–68.7% and PFS up to 33.8%–48% (8–11), which are slightly better than the results in our study. This is probably because the median tumor size in most of these studies was around 5 cm, whereas the tumor volume in our study was relatively large, with a median tumor diameter of 8.4 cm. C.L Chiang (27) retrospectively evaluated the efficacy of TACE combined with SBRT as initial therapy in BCLC stage B-C HCC. The median prescription dose in an equivalent dose of 2 Gy per fraction (EQD2, α/β = 10) was 37.3 Gy, and BED10 was 44.76 Gy. The median OS was 19.8 months. Subgroup analysis found that the median OS of BCLC stage B patients was 25.7 months, and the median PFS was 9.1 months (95% CI, 7.2–19.8). The OS in our study seems more favorable than that in the above study, which may be due to the higher prescription dose in our study (100 Gy for mBED10). Higher effective biological dose might result in better LC and long-term survival rate.

Although 30%–40% of primary liver cancer is negative for AFP (29), it is still a sensitive tumor marker for the detection of HCC and a useful predictive factor due to its specificity for patient survival after locoregional or systemic treatment in HCC (30, 31). A South Korean study reported that AFP normalization within 3 months after SBRT was a prognostic surrogate for OS and PFS in patients with small HCC (32). The prognostic value of AFP normalization after SBRT is still unknown due to the lack of randomized controlled studies and several other factors. First, there is no uniform standard of the optimal decrement of the AFP level. Second, the cut-off value of pretreatment AFP that would be adequate in order to apply AFP normalization as a surrogate is still unknown. In this study, we found that pretreatment AFP level ≥ 200 ng/ml was an independent adverse prognostic factor for OS. In addition, we conducted a subgroup analysis, which indicated that within 3 months after CyberKnife SBRT, the OS was significantly increased in patients whose AFP decreased by more than 75% compared with those whose AFP decreased by less than 75%. Moreover, the difference was statistically significant (P = 0.018). This result was consistent with previous reports of Erhua Yao (33).

With regard to the side effects, TACE combined with CyberKnife SBRT was well tolerated, with the majority of adverse effects being grade 1 and 2. No patient exhibited classic RILD, but one patient had liver transaminase elevation without disease progression, which is defined as non-classic RILD. One patient developed gastric ulcer, whose target lesion was adjacent to the stomach. The maximum point dose reached 7 Gy*5fx, which might exceed the tolerance of the stomach. The toxic reaction could be gradually repaired after symptomatic treatment. The treatment-related adverse effects for most patients were tolerable, which may be due to the fact that 82.5% of the patients in this study had a Child–Pugh score of A. In addition, the interval between the last TACE and CyberKnife was more than 3 weeks, exceeding the time window of liver function repair, which was conducive to the recovery of liver function. Consistent with the opinion of most previous studies, TACE combined with SBRT was well tolerated in the treatment of HCC patients with Child–Pugh score of A/B7 (8). However, Lasley (34) found that the hepatotoxicity of patients with Child–Pugh score of B was increased compared with that of patients with Child–Pugh score of A after SBRT, suggesting that the use of this treatment should be cautioned in patients with Child–Pugh score of B. The latest technology, MRI-based radiotherapy can provide real-time visualization of both the tumor and nearby organs, potentially reduce toxicity to critical structures. Superior to CT-guided radiotherapy, this technology has the potential to define tolerances to gastrointestinal and hepatobiliary structures, which might increase the number of patients eligible for high-dose ablative liver radiotherapy (35).

This study has several limitations. First, this is a retrospective cohort study from a single SBRT center, and the results might not be generalizable. Second, the number of enrolled patients is relatively small. Further randomized controlled studies are needed to address the limitations and determine the intended population, the most appropriate time for SBRT, the optimal number of TACE before SBRT, and the formulation of prescription dose and fractions in combination therapy. Recently, a randomized phase 3 trial (IMbrave150) showed that atezolizumab plus bevacizumab can prolong OS and PFS than sorafenib in patients with advanced unresectable BCLC B-C HCC (36). Immunotherapy has become the new standard of care for advanced HCC. Further studies are warranted to investigate the optimal algorithm of these therapeutic options in advanced unresectable HCC.

## Conclusion

In conclusion, treatment with TACE plus CyberKnife SBRT was associated with optimal efficacy and acceptable toxicity in patients with unresectable BCLC B HCC.

## Data Availability Statement

The original contributions presented in the study are included in the article/supplementary material. Further inquiries can be directed to the corresponding author.

## Ethics Statement

The studies involving human participants were reviewed and approved by Jinling Hospital review board. The patients/participants provided their written informed consent to participate in this study.

## Author Contributions

CJ: project design, data collection, assembly, analysis, manuscript writing. SJ: data collection and assembly. HZ: data collection and assembly. AL: Investigation. XQ: data collection and assembly. XZ: project design. ZS: project conception and design, data interpretation. All authors contributed to the article and approved the submitted version.

## Funding

This work was supported by grants from: 1. The Nanjing Municipal Science and Technology Committee of Jiangsu Province, China (grant number: 201803050). 2. The Jiangsu Post-doctoral Research Funding Program, China (grant number: 2020Z305).

## Conflict of Interest

The authors declare that the research was conducted in the absence of any commercial or financial relationships that could be construed as a potential conflict of interest.
